# Supplementary material for: Meta-Analysis and Gene Set Enrichment Relative to ER Status Reveal Elevated Activity of MYC and E2F in the “Basal” Breast Cancer Subgroup
Source: PLoS One. 2009 Mar 9;4(3):e4710. doi: 10.1371/journal.pone.0004710 (PMC2650420; doi:10.1371/journal.pone.0004710)
Supplement: Dataset S1 — GSEA results from first screen. The file contains a zipped archive of the HTML results of the second GSEA screen. Reports for enriched or depleted datasets may be accessed from the “index.html” link within the my_analysis.GseaPreranked.1219975950625.rpt.zip ZIP archive. Enrichment plots are available for each of the 50 most enriched and depleted gene sets. (13.32 MB ZIP) [file pone.0004710.s001.zip › my_analysis.GseaPreranked.1219975950625.rpt.zip!//AGUIRRE_PANCREAS_CHR12.html]

Details for gene set AGUIRRE\_PANCREAS\_CHR12[GSEA]

|  || Dataset | zSco.ERneg.to.ERpos\_collapsed |
| Phenotype | NoPhenotypeAvailable |
| Upregulated in class | na\_neg |
| GeneSet | AGUIRRE\_PANCREAS\_CHR12 |
| Enrichment Score (ES) | -0.5822234 |
| Normalized Enrichment Score (NES) | -2.4281342 |
| Nominal p-value | 0.0 |
| FDR q-value | 0.0 |
| FWER p-Value | 0.0 |
Table: GSEA Results Summary

  

Fig 1: Enrichment plot: AGUIRRE\_PANCREAS\_CHR12      
 Profile of the Running ES Score & Positions of GeneSet Members on the Rank Ordered List

  

| PROBE | GENE SYMBOL | GENE\_TITLE | RANK IN GENE LIST | RANK METRIC SCORE | RUNNING ES | CORE ENRICHMENT || 1 | OR7E47P |  |  | 783 | 3.794 | -0.0316 | No |
| 2 | CCDC59 |  |  | 1384 | 3.055 | -0.0547 | No |
| 3 | XPOT |  |  | 1517 | 2.930 | -0.0435 | No |
| 4 | CMAS |  |  | 1800 | 2.690 | -0.0453 | No |
| 5 | ACVR1B |  |  | 2189 | 2.393 | -0.0572 | No |
| 6 | PLEKHA9 /// PLEKHA8 |  |  | 3679 | 1.628 | -0.1577 | No |
| 7 | LOC57228 |  |  | 3794 | 1.588 | -0.1548 | No |
| 8 | RACGAP1 |  |  | 4097 | 1.475 | -0.1669 | No |
| 9 | SLC11A2 |  |  | 4406 | 1.353 | -0.1804 | No |
| 10 | BTG1 |  |  | 4412 | 1.350 | -0.1710 | No |
| 11 | DYRK2 |  |  | 6103 | 0.782 | -0.2928 | No |
| 12 | FKBP11 |  |  | 6214 | 0.746 | -0.2957 | No |
| 13 | RARG |  |  | 7070 | 0.461 | -0.3568 | No |
| 14 | HOXC11 |  |  | 7294 | 0.385 | -0.3708 | No |
| 15 | PPP1R12A |  |  | 7534 | 0.295 | -0.3867 | No |
| 16 | KITLG |  |  | 7921 | 0.138 | -0.4148 | No |
| 17 | VDR |  |  | 7985 | 0.110 | -0.4188 | No |
| 18 | CCT2 |  |  | 8514 | -0.085 | -0.4580 | No |
| 19 | ELA2A |  |  | 9197 | -0.404 | -0.5065 | No |
| 20 | ATP2B1 |  |  | 9376 | -0.495 | -0.5163 | No |
| 21 | TFCP2 |  |  | 9836 | -0.747 | -0.5455 | No |
| 22 | LEMD3 |  |  | 9918 | -0.793 | -0.5459 | No |
| 23 | SFRS2IP |  |  | 10128 | -0.919 | -0.5550 | No |
| 24 | PTK9 |  |  | 10177 | -0.943 | -0.5518 | No |
| 25 | GNS |  |  | 10327 | -1.046 | -0.5555 | No |
| 26 | HNRPA1 |  |  | 10609 | -1.250 | -0.5676 | No |
| 27 | HNRPA1 /// RP11-78J2 |  |  | 10660 | -1.291 | -0.5620 | No |
| 28 | DUSP6 |  |  | 10680 | -1.305 | -0.5540 | No |
| 29 | ARF3 |  |  | 11055 | -1.622 | -0.5705 | Yes |
| 30 | FLJ20489 |  |  | 11097 | -1.660 | -0.5616 | Yes |
| 31 | SPATS2 |  |  | 11318 | -1.841 | -0.5648 | Yes |
| 32 | YEATS4 |  |  | 11327 | -1.848 | -0.5521 | Yes |
| 33 | C12ORF29 |  |  | 11500 | -2.037 | -0.5503 | Yes |
| 34 | CACNB3 |  |  | 11624 | -2.146 | -0.5441 | Yes |
| 35 | HNRPA1 /// LOC644245 |  |  | 11746 | -2.262 | -0.5368 | Yes |
| 36 | LMBR1L |  |  | 11758 | -2.274 | -0.5212 | Yes |
| 37 | CAND1 |  |  | 11831 | -2.352 | -0.5096 | Yes |
| 38 | MYL6 |  |  | 11843 | -2.362 | -0.4933 | Yes |
| 39 | EIF4B |  |  | 11934 | -2.480 | -0.4822 | Yes |
| 40 | TBK1 |  |  | 12180 | -2.817 | -0.4803 | Yes |
| 41 | DAZAP2 |  |  | 12274 | -2.932 | -0.4661 | Yes |
| 42 | CEP290 |  |  | 12291 | -2.957 | -0.4459 | Yes |
| 43 | ORMDL2 |  |  | 12304 | -2.969 | -0.4253 | Yes |
| 44 | RAP1B /// LOC643752 |  |  | 12350 | -3.023 | -0.4068 | Yes |
| 45 | TEGT |  |  | 12420 | -3.138 | -0.3893 | Yes |
| 46 | HOXC4 |  |  | 12463 | -3.204 | -0.3693 | Yes |
| 47 | SMUG1 |  |  | 12520 | -3.294 | -0.3497 | Yes |
| 48 | PFDN5 |  |  | 12640 | -3.509 | -0.3333 | Yes |
| 49 | MCRS1 |  |  | 12800 | -3.891 | -0.3171 | Yes |
| 50 | SLC35E3 |  |  | 12801 | -3.893 | -0.2890 | Yes |
| 51 | CD63 |  |  | 12872 | -4.071 | -0.2648 | Yes |
| 52 | C12ORF41 |  |  | 12903 | -4.160 | -0.2370 | Yes |
| 53 | COPZ1 |  |  | 13011 | -4.495 | -0.2125 | Yes |
| 54 | BLOC1S1 |  |  | 13032 | -4.559 | -0.1810 | Yes |
| 55 | DDX23 |  |  | 13079 | -4.749 | -0.1501 | Yes |
| 56 | PRKAG1 |  |  | 13118 | -4.904 | -0.1175 | Yes |
| 57 | C12ORF10 |  |  | 13236 | -5.737 | -0.0848 | Yes |
| 58 | TMBIM4 |  |  | 13262 | -6.100 | -0.0426 | Yes |
| 59 | MFSD5 |  |  | 13293 | -6.481 | 0.0020 | Yes |
Table: GSEA details [plain text format]

  

Fig 2: AGUIRRE\_PANCREAS\_CHR12: Random ES distribution      
 Gene set null distribution of ES for **AGUIRRE\_PANCREAS\_CHR12**
